# Non-typhoidal *Salmonella* serovars in poultry farms in central Ethiopia: prevalence and antimicrobial resistance

**DOI:** 10.1186/s12917-018-1539-4

**Published:** 2018-07-06

**Authors:** Tadesse Eguale

**Affiliations:** Aklilu Lemma Institute of Pathobiology, Addis Ababa University, P.O. Box 1176, Addis Ababa, Ethiopia

**Keywords:** Poultry, Non-typhoidal *Salmonella*, Antimicrobial resistance, Prevalence, Ethiopia

## Abstract

**Background:**

Poultry is one of the common sources of non-typhoidal *Salmonella* and poultry products are the major sources of human infection with non-typhoidal *Salmonella.* In spite of flourishing poultry industry in the country, data on prevalence and antimicrobial susceptibility of non-typhoidal *Salmonella* serovars at farm level is not available in Ethiopia. This study investigated prevalence, serotype distribution and antimicrobial resistance of non-typhoidal *Salmonella* in poultry farms in Addis Ababa and its surrounding districts.

**Results:**

A total of 549 fresh pool of fecal droppings (*n* = 3 each) were collected from 48 poultry farms and cultured for *Salmonella* using standard laboratory technique and serotyped using slide agglutination technique. Susceptibility of *Salmonella* isolates to18 antimicrobials was tested according to CLSI guideline using Kirby-Bauer disk diffusion assay. *Salmonella* was recovered in 7 (14.6%) of the farms and 26 (4.7%) of the samples. *Salmonella* was more common in poultry farms with larger flock size than in the smaller ones and in Ada’a district as compared to other districts. All isolates were obtained from farms containing layers. Two out of 6 (33.3%) farms that kept birds in cage were positive for *Salmonella* while only 5 (11.9%) of the 42 farms who used floor system were positive. Oxytetracycline was used widely in 40 (83.3%) of the farms, followed by amoxicillin 14 (29.2%) and sulfonamides 11 (22.9%). *Salmonella* Saintpaul was the dominant serotype detected accounting for 20 (76.9%) of all isolates. Other serovars, such as *S.* Typhimurium3 (11.5%), *S.* Kentucky 2 (7.7%) and *S.* Haifa 1 (3.8%) were also detected. Of all the *Salmonella* isolates tested, 24 (92.3%) were intermediately or fully resistant to sulfisoxazole and streptomycin, 12 (46.2%) to cephalothin, while 11 (42.3%) were resistant to ampicillin, amoxicillin+clavulanic acid, kanamycin and chloramphenicol. Multidrug resistance (MDR) to several drugs was common in *S.* Kentucky and *S.* Saintpaul.

**Conclusion:**

Despite low prevalence of *Salmonella* in poultry farms in the study area, circulation of MDR strains in some farms warrant special biosecurity measures to hinder dissemination of these pathogens to other farms and the public. Moreover, awareness creation on prudent use of antimicrobials is recommended.

## Background

*Salmonella* is one of the major causes of food-borne diseases worldwide [1]. Poultry and other food animals are considered the common reservoirs of *Salmonella enterica* and undercooked poultry products are the major sources of human infection with non-typhoidal *Salmonella* [2, 3]*.* Several host unrestricted *S. enterica* serovars frequently isolated from poultry without showing any clinical signs usually infect a wider range of hosts and cause disease in humans as well [4].

It has been shown that some of the most commonly detected serovars in chickens in a given geographic area are also among the top serovars associated with human infections indicating the role of *Salmonella* colonization of poultry farms to public health [5]. Knowledge on distribution of *Salmonella* serovars in food animals and humans is useful to understand the trends of *Salmonella* epidemiology and to identify serovars that cluster over time and space. Temporal and spatial variation in rate and distribution of *Salmonella* serovars in poultry industry has been reported [2, 6].

Developed countries conduct routine surveillance of *Salmonella* in poultry farms to understand the level of colonization by *Salmonella*, serovars involved and drug resistance profile with the aim of designing ways of reducing public health salmonellosis of poultry origin [7, 8]. However, in developing countries like Ethiopia, little effort is made to monitor *Salmonella* in poultry farms and information on prevalence and serotype distribution as well as phenotypic and genotypic relatedness of *Salmonella* isolated from poultry and humans is not well documented. Local knowledge on prevalence of *Salmonella*, serotype distribution and associated risk factors is important to implement appropriate control strategy to reduce wider dissemination of important zoonotic serovars [2].

There is little available literature on farm level prevalence and serotype distribution of non-typhoidal *Salmonella* in poultry farms in Ethiopia. Previous studies conducted on retail raw chicken products reported 17.9% prevalence of *Salmonella*, the dominant serovars being *S*. Braenderup (31.5%), *S.* Anatum (25.9%), *S.* Saintpaul (14.8%) and *S.* Uganda (11.1%) [9]. Another study also reported that 14% of chicken carcass from supermarkets in Addis Ababa were positive for *Salmonella. S.* Braenderup (41.4%), *S.* Hadar (20.7%), *S.* Newprt (13.8%) and *S.* Typhimurium (10.3%) were the dominant serovars detected in poultry products in Addis Ababa [10]. However, source of *Salmonella* contamination in these poultry products could be either from farm or due to cross contamination during slaughter, transportation or storage. Recent study in southern Ethiopia showed that 16.7% of samples from poultry and the environment of three poultry farms were positive for *Salmonella* although this study did not show whether *Salmonella* isolates were host specific *Salmonella* serovars or host unrestricted non-typhoidal *Salmonella* serovars [11].

Majority of the *Salmonella* isolates from poultry products and poultry farms in the previous studies were found to be resistant to several antimicrobials. Information on farm level prevalence and antimicrobial susceptibility status of isolates can explain the level of public health risk associated with poultry products. The aim of this study was therefore to determine the prevalence, serotype distribution and antimicrobial resistance of *salmonella* in poultry farms in central Ethiopia. The type of antimicrobials and disinfectants commonly employed in poultry farms were also assessed.

## Methods

### Study design, study area and study animals

A cross-sectional study was conducted in Addis Ababa and 3 districts of Oromia region located at the outskirt of Addis Ababa from July 2013–January 2014. A total of 549 pooled fresh fecal droppings (from 3 chicken each) were collected in 48 farms (Ada’a district *n* = 33, Addis Ababa *n* = 6, Sebeta *n* = 6, Barake *n* = 3). Inclusion of farms in the sampling was based on representation of the area under study, willingness of the owners, availability of poultry farms in the study area, and the flock having a minimum of 50 birds. Most of the poultry farms investigated in the current study were those from Ada’a district due to large number of poultry farms in this district.

### Data and sample collection

Information such as type of poultry farm, whether it is broiler or layer, flock size, birds housing system, age of birds, purpose and types of antimicrobials and disinfectants commonly used in the farm during the last 6 months were recorded using a purposively designed questionnaire. Collection of data was performed at the time of fecal sample collection from each farm. Pooled fresh fecal droppings(from 3 chickens) were collected using clean disposable gloves in to sterile zippered plastic bags which were transported to microbiology laboratory of Aklilu Lemma Institute of Pathobiology, Addis Ababa University in an ice box within 3–4 h of collection.

### *Salmonella* isolation, identification, serotyping and phage typing

*Salmonella* isolation and identification was carried out using conventional methods [12, 13]. Briefly, fresh fecal droppings from three chicken was thoroughly mixed of which 10 g of feces was suspended in 90 ml of buffered peptone water (BPW) (Becton Dickinson, Sparks, MD) and incubated overnight at 37 °C. Enrichment, culturing on selective media, and biochemical analysis of presumptive *Salmonella* colonies was conducted as shown previously [14]. Genus specific PCR was used to confirm isolates suspected to be *Salmonella* by biochemical tests [15]. *Salmonella* Typhimurium (ATCC 14028) was used as a positive control during biochemical analysis and PCR. Confirmed *Salmonella* isolates were stored at − 80 °C in 20% glycerol till further investigation.

Serotyping and phage typing of *Salmonella* isolates was conducted at the World Organization for Animal Health (OIÉ) Reference Laboratory for salmonellosis, Public Health Agency of Canada’s National Microbiology at Guelph. Determination of serovars was conducted using serum agglutination technique as shown previously [16, 17], based on identification of somatic (O) antigens [18] and flagellar (H) antigens [19].

### Antimicrobial susceptibility testing

Isolates were investigated for susceptibility to 18 antimicrobials using the Kirby-Bauer disk diffusion method according to Clinical and Laboratory Standards Institute guidelines [20]. Antimicrobials used in the current study were amikacin (30 μg), amoxicillin + clavulanic acid (20/10 μg), ampicillin (10 μg), cefoxitin (30 μg), ceftriaxone (30 μg), cephalothin (30 μg), chloramphenicol (30 μg), ciprofloxacin (5 μg), gentamicin (10 μg), kanamycin (30 μg), nalidixic acid (30 μg), neomycin (30 μg), nitrofurantoin (100 μg), streptomycin (10 μg), sulfisoxazole (1000 μg), sulfamethoxazole + trimethoprim (23.75/1.25 μg), trimethoprim (5 μg) and tetracycline (30 μg). All of them were from Sensi-Discs, Becton, Dickinson and Company, Loveton, USA. The interpretation cut off points for susceptibility status of isolates was based on the CLSI guidelines [20]. For the purpose of analysis, all readings classified as intermediate were considered as resistant unless indicated. *E. coli* ATCC 25922 was used as a quality control organism.

### Statistical analysis

Sample level prevalence of *Salmonella* was calculated as percentage of *Salmonella* culture positive fecal samples among total number of samples examined. Farm level prevalence was calculated as the percentage of farms with one or more *Salmonella* culture positive pooled fecal sample among the total farms sampled. Association of *Salmonella* detection with various factors was tested using exact test and *p*-value < 0.05 was considered significant.

## Results

### Farm level *Salmonella* occurence with respect to various factors

*Salmonella* was isolated from 14.6% (7/48) of poultry farms with individual sample level prevalence of 4.7% (Table 1). *Salmonella* was more common in poultry farms with larger flock size and in age group of 2–6 months (Table 2). Majority of the farms studied contained layers or young pullets grown for egg production (*n* = 43, 89.6%); whereas only (*n* = 5; 11.4%) were keeping broilers. *Salmonella* was not detected from the broiler farms. *Salmonella* isolation was also more common in farms of the Ada’a district as compared to other districts. Majority of the farms (*n* = 42; 87.5%) keep their birds on floor system and 12.5% (6/48) use cage system. Out of the farms that use cage system 33.3% (2/6) were positive for *Salmonella* whereas 11.9% (5/42) of farms that use floor system were found positive for *Salmonella*.

**Table 1:** Prevalence of *Salmonella* in poultry farms in Addis Ababa and surrounding districts

	No. of farms	Average no. of birds /farm	No. of samples^a^	No. positive samples	% positive samples	(%) positive farms
Ada’a	33	4638	464	25	5.4	18.2
Addis Ababa	6	1075	45	1	2.2	16.7
Barake	3	395	18	0	0	0
Sebeta	6	627	22	0	0	0
Total	48	1684	549	26	4.7	14.6

^a^Samples were pool of fecal droppings from 3 chicken

**Table 2:** Occurrence of *Salmonella* in poultry farms stratified by selected factors

Selected Factors	No. of farms	No. of *Salmonella* positive farms	% of farms positive for *Salmonella*	*p*-value*
Commodity type				
Layers	43	7	16.3	1.000
Broilers	5	0	0	
Use of disinfectants				
Yes	26	6	23.1	0.106
No	22	1	4.5	
Age of birds in months				
< 2	8	0	0	0.608
2–6	17	4	24	
7–12	13	2	15.4	
> 12	10	1	10	
Flock size				
< 1000(Small)	22	2	9.1	0.648
1000–5000(Medium)	17	3	17.7	
> 5000(Large)	9	2	22.2	
Poultry housing system				
Floor	42	5	11.9	0.206
Cage	6	2	33.3	

*Exact test was used to obtain *p*-value

### Antimicrobials used in poultry farms

Among the common antimicrobials, oxytetracycline was used widely in 40 (83.3%) of the farms, followed by amoxicillin (29.2%) and sulfonamides (22.9%). Other antimicrobials such as fluoroquinolones (enrofloxacin and ciprofloxacin), and florfenicol were also used in 11 (22.9%) and 7 (14.6%) of the farms respectively, whereas 6(12.5%) of the farms reported that they did not use any antimicrobials during last 6 months. None of the farms reported use of antimicrobials as feed additive. All of the farms use antimicrobials for therapeutic or prophylactic purposes when there is one or more sick birds in the flock. Interestingly, in one of the poultry farms in Adaa district, the use of human preparation of ciprofloxacin tablet was observed. *Salmonella* was recovered more frequently in farms which use only amoxicillin, sulfadimidine and oxytetracycline than those farms which use fluoroquinolones and florfenicol. Recent use of antimicrobials and occurrence of *Salmonella* in farms is shown in Table 3. All samples from six farms with no history of use of antimicrobials were also not culture positive for *Salmonella.* Twenty-three (47.9%) of the farms reported use of sodium hypochlorite disinfectant as foot bath, for cleaning poultry houses before introduction of new stock and to clean feeding utensils, while 4(8.3%) of the farms used copper sulfate. The remaining 21(43.8%) of the poultry farms were not using any disinfectant.

**Table 3:** Recent use of antimicrobials and occurrence of *Salmonella* in poultry farms

Type of Antimicrobials used during the last 6 months	No. of farms	No. of *Salmonella* positive farms	% of farms positive for *Salmonella*
Amoxicillin only	2	0	0
Oxytetracyline only	18	4	22.2
Oxytetracycline + ciprofloxacin	3	0	0
Oxytetracycline + florfenicol + enrofloxacin	4	0	0
Oxytetracycline + sulfonamides	3	1	33.3
Oxytetracycline + amoxicillin	4	1	25
Oxytetracycline + sulfonamides + amoxicillin	8	1	12.5
Did not use antimicrobial agent	6	0	0

### *Salmonella* serotype distribution and antimicrobial susceptibility

*Salmonella* Saintpaul was the dominant serotype detected in poultry farms accounting for 20 (76.9%) of all isolates. Other serotypes, such as *S.* Typhimurium (*n* = 3), *S.* Kentucky (*n* = 2) and *S.* Haifa (*n* = 1) were also detected. Rate of resistance to antimicrobials tested and resistance patterns of the isolates are shown in Tables 4 and 5 respectively. Of all the *Salmonella* isolates tested, (*n* = 24, 92.3%) were resistant to sulfisoxazole and streptomycin, (*n* = 12, 46.2%) of the isolates were resistant to cephalothin, while (*n* = 11, 42.3%) were resistant to ampicillin, amoxicillin + clavulanic acid, kanamycin and chloramphenicol (Table 4).

**Table 4:** *Salmonella* serovar distribution and rate of resistance to antimicrobial agents

Antimicrobial agents	*Salmonella* serovars and resistance rate^a^				Total No. (%) resistant
	*S*. Saintpaul (*n* = 20)	*S*. Typhimurium (*n* = 3)	*S*. Kentucky (*n* = 2)	*S*. Haifa (*n* = 1)	
	No. resistant (%)	No. resistant (%)	No. resistant (%)	No. resistant (%)	
Ampicillin	9 (45)	0	2 (100)	0	11 (42.3)
Amoxicillin+clavulanic acid	9 (45)	0	2 (100)	0	11 (42.3)
Chloramphenicol	10 (50)	0	1 (50)	0	11 (42.3)
Cephlothin	10 (50)	0	2 (100)	0	12 (46.2)
Ciprofloxacin	0	0	2 (100)	0	2 (7.7)
Cefoxitin	0	0	0	0	0
Gentamicin	0	0	2 (100)	0	2 (7.7)
Kanamycin	8 (40)	2 (66.7)	0	1 (100)	11 (42.3)
Sulfamethoxazole+trimethoprim	0	0	0	1 (100)	1 (3.9)
Trimethoprim	0	0	0	1 (100)	1 (3.9)
Tetracycline	4 (20)	1 (33.3)	2 (100)	1 (100)	8 (30.8)
Sulfisoxazole	18 (90)	3 (100)	2 (100)	1 (100)	24 (92.3)
Streptomycin	18 (90)	3 (100)	2 (100)	1 (100)	24 (92.3)
Nitrofurantoin	5 (25)	1 (33.3)	0	1 (100)	7 (26.7)
Nalidixic acid	2 (10)	0	2 (100)	1 (100)	5 (19
Neomycin	3 (15)	0	0	0	3 (11.5)

^a^Isolates with intermediate susceptibility were also considered resistant for this analysis

**Table 5:** *Salmonella* serotypes isolated from poultry farms and their antimicrobial resistance pattern

No.	Study site	Farm Code	Isolate code	Serotype	Resistance pattern	
					Intermediate	Resistant
1	Adaa	DZP-20	DP-213 T	Kentucky	C	Amp,Amc,Cf,Cip,Gm,Te,Su,S,Na
2	Adaa	DZP-20	DP-220 T	Kentucky	–	Amp,Amc,Cf,Cip,Gm,Te,Su,S,Na
3	Addis Ababa	AAP-08	AP-H2O	Haifa	K,S	Sxt,Tmp,Te,Su,Nitro,Na
4	Adaa	DZP-03	DP-23 T	Saintpaul	Su	–
5	Adaa	DZP-03	Dp-24 T	Saintpaul	Su,S	–
6	Adaa	DZP-03	Dp-25R	Saintpaul	SuS	–
7	Adaa	DZP-03	DP-26R	Saintpaul	Cip,Su,SNitro,N	–
8	Adaa	DZP-03	DP-27R	Saintpaul	Su,S	–
9	Adaa	DZP-11	DP-116 T	Typhimurium	K	Te,Su,S
10	Adaa	DZP-08	DP-70 T	Typhimurium	SuS	–
11	Adaa	DZP-08	DP-71 T	Typhimurium	K,Su,S,Nitro	–
12	Adaa	DZP-33	DP-107	Saintpaul	Amc,Cf,K,S	Amp,C,Te,Su
13	Adaa	DZP-33	DP-117	Saintpaul	Amc,Cf,K,S	Amp,C,Su
14	Adaa	DZP-33	DP-128	Saintpaul	-	-
15	Adaa	DZP-33	DP-131	Saintpaul	K,S	–
16	Adaa	DZP-33	DP-110	Saintpaul	Amc,Cf,S	Amp,C,Te,Su
17	Adaa	DZP-33	DP-114	Saintpaul	SuS	
18	Adaa	DZP-33	DP-126	Saintpaul	Cf,S	Amp,Amc,C,Su
19	Adaa	DZP-12	DP-313	Saintpaul	S,K	Amp,Amc,C,Cf,Te,Su
20	Adaa	DZP-12	DP-325	Saintpaul	Amc,Cf,Su,S,Nitro	Amp,Amc,C,Cf,Su,S,Nitro,Na
21	Adaa	DZP-12	DP-327	Saintpaul	K,S,Nitro	Su
22	Adaa	DZP-12	DP-328	Saintpaul	Amc,Cip,S,N	Amp,C,Cf,Te,Su
23	Adaa	DZP-12	DP-339	Saintpaul	K,Su,S	–
24	Adaa	DZP-12	DP-322	Saintpaul	Amc,Cf, Su,S,Nitro	Amp,Amc,C,Cf,Su,S,Nitro,Na
25	Adaa	DZP-12	DP-326	Saintpaul	Cf,S	Amp,Amc,C,Su
26	Adaa	DZP-12	DP-308	Saintpaul	Amc,Cip,K,Su,S,Na,N	Amp,C,Cf,Nitro

*Amp* ampicillin, *Amc* amoxicillin and clavulanic acid, *Cf* cephalothin, *Cip* ciprofloxacin, *Gm* gentamicin, *K* kanamycin, *Tmp* trimethoprim, *Te* tetracycline, *Su* sulfisoxazole, *S* streptomycin, *Nitro* nitrofurantoin, *Na* nalidixic acid, *N* neomycin, -sensitive

Overall, multidrug resistance was commonly detected in *Salmonella* isolates in the current study particularly in strains belonging to *S*. Saintpaul and the two *S*. Kentucky isolates. All *S.* Saintpaul strains in the current study were isolated from farms in Ada’a district. However, there was wide diversity in their antimicrobial susceptibility pattern even among isolates obtained from the same farm. Some of them were resistant to only few antimicrobials while others were MDR to several antimicrobials. The two *S.* Kentucky isolates were resistant to 9 of the 18 antimicrobials tested (Table 5).

## Discussion

Colonization of poultry with *Salmonella* without detectable clinical signs at farm level followed by contamination of poultry products with subsequent access to human food chain has been considered as the major sources of human salmonellosis [21, 22]. *Salmonella* in healthy poultry is the main risk factor for possible outbreak of human salmonellosis and epidemiological studies have shown the huge contribution of contaminated poultry products to human salmonellosis [23, 24]. In fact, some countries have shown that successful control measures involving surveillance, improved biosecurity and vaccination targeting specific serovars in poultry can result in reduction of human salmonellosis cases [21, 24].

In the current study, 7(14.6%) of the 48 examined poultry farms were positive for *Salmonella*. This is very much low compared to studies conducted in Morocco and Nigeria where 76.7% and [25], 43.6% [26] of the poultry farms were contaminated by *Salmonella,* respectively. Sample level prevalence of *Salmonella* was also low in the current study (4.7%) compared to previous studies conducted elsewhere. For instance, *Salmonella* prevalence in fecal samples from conventional poultry farms in USA was reported to be 38.8% while it was 5.6% in organic farms [27]. *Salmonella* prevalence in conventional poultry is usually very high in different countries [28–31]. The possible reason for low prevalence of *Salmonella* in the current study could be due to the fact that most of the poultry farms in the current study were small scale farms holding small number of birds unlike most of the large commercial poultry farms where they keep thousands of birds and the feeding and management activities associated with intensification allows easy dissemination of the pathogen within the farm. This finding is in agreement with previous report where large farms were significantly associated with high prevalence of *Salmonella* compared to medium and small farms [32].

Both farm level and pooled sample level prevalence of *Salmonella* was high in farms from Ada’a district compared to other areas, which could be due to larger number of poultry farms examined from this district compared to others as well as difference in agroecology. Ada’a district is highly concentrated with large number of poultry farms and is located in rift valley which is relatively warm region compared to Addis Ababa, Sebeta and Barake districts. The fact that most of the large poultry farms in the country including the parent stocks are located in Ada’a district and most of the farms from this area shared a single serotype, *S.* Saintpaul implies the possibility of transmission of *Salmonella* from farm to farm in this town. *Salmonella* Saintpaul is not frequently isolated from poultry in other previous studies elsewhere. *Salmonella* Kentucky was the dominant serovar in studies conducted in Nigeria [26] and Bangladesh [31] and *S.* Entertidis was dominant in Spain [33]; while *S*. Typhimurium was dominant in China [34]. Although there is no serotype data on *Salmonella* isolates from poultry at farm level in Ethiopia, previous study from poultry food items in Addis Ababa did not report *S*. Saintpaul [35]. As most of the farms obtain their day old chickens or pullets from a few parent stock farms located in this district, there is likelihood of contamination of poultry from source farms. In addition, *S.* Saintpaul was the major serotype detected in dairy farms in this study area which suggests possibility of transmission between dairy and poultry farms [14].

The high rate of resistance to sulfixazole and streptomycin (92.3%) is not concordant with the current rate of use of antimicrobials in farms investigated. However, previously, different sulfonamide drugs and streptomycin together with penicillin were the common antimicrobials frequently used in the country for treatment of various infectious diseases in veterinary medicine and recent studies showed that sulfonamides and streptomycin are the 2nd and 3rd most prescribed veterinary medications respectively in the study area next to oxytetracycline [36]. Similarly, high resistance rate to ampicillin and tetracycline could be due to long term use of these antimicrobials in veterinary medicine including poultry. Interestingly, the two *S*. Kentucky isolates resistant to several drugs including nalidixic acid and ciprofloxacin were isolated from one of a few farms which reported use of fluoroquinolones for therapeutic purposes in the farm suggesting possible contribution of use of these drugs in the farm for selection of these strains. Eleven (42.3%) of the isolates in the current study, most of which belonging to *S*. Saintpaul from farms in Ada’a district were resistant to chloramphenicol unlike previous study where all of the isolates obtained from food of animal origin including poultry products were fully susceptible to chloramphenicol [35]. Unlike previous study in south Ethiopia [11] where extremely high proportion of *Salmonella* isolates (97.8%) were resistant to second generation cephalosporin (cefoxitin), in this study, none of the isolates were resistant to this drug. This could be due to over use of betalactam drugs in the previous farms.

## Conclusion

Despite low prevalence of *Salmonella* in poultry farms in the study area, circulation of MDR strains in some farms warrant special biosecurity measures to hinder dissemination of these pathogens to other farms and the public. Moreover, awareness creation on prudent use of antimicrobials is recommended.

## Abbreviations

BPW: Buffered peptone water
MDR: Multi-drug resistance
RVB: Rappaport-vassiliadis broth
TTB: Tetrathionate broth
XLT-4: Xylose lysine tergitol 4
